# Nitro­sonium tetra­fluorido­borate, NOBF_4_

**DOI:** 10.1107/S2414314621012153

**Published:** 2021-11-18

**Authors:** Matic Lozinšek

**Affiliations:** a Department of Inorganic Chemistry and Technology, Jožef Stefan Institute, Jamova cesta 39, 1000 Ljubljana, Slovenia; Vienna University of Technology, Austria

**Keywords:** crystal structure, tetra­fluorido­borate, nitro­sonium, redetermination

## Abstract

The crystal structure of oxido­nitro­gen(1+) tetra­fluorido­borate (nitro­sonium tetra­fluorido­borate), NO^+^BF_4_^−^, was refined on the basis of single-crystal X-ray diffraction data at 150 K. The compound crystallizes in the baryte structure type with ortho­rhom­bic *Pnma* symmetry. The crystal structure exhibits cationic disorder with equal occupation of N and O atoms at the same site.

## Abstract 2

NOBF_4_ crystallizes in the baryte structure type with ortho­rhom­bic *Pnma* symmetry and exhibits cationic disorder.

## Graphical abstract

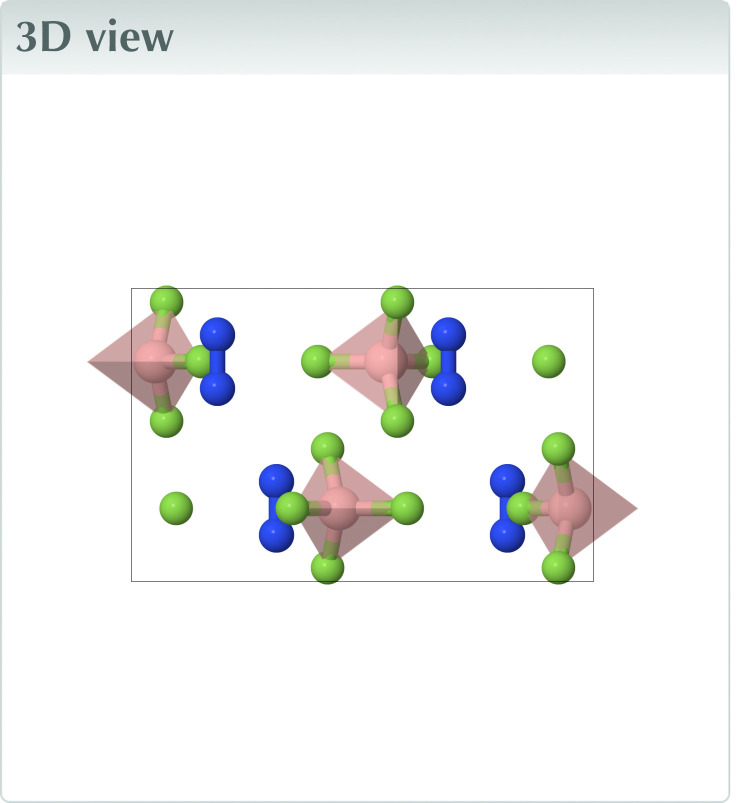

## Structure description

Numerous nitro­sonium fluorido salts are known (*e.g.*, Sunder *et al.*, 1979 ▸; Mazej *et al.*, 2009 ▸) and several of them have been structurally characterized (*e.g.*, Adam *et al.*, 1996 ▸). Nonetheless, for nitro­sonium tetra­fluorido­borate (NOBF_4_), which is an efficient one-electron oxidant, nitro­sating and diazo­tizing agent (Olah *et al.*, 2004 ▸), only the unit-cell parameters derived from X-ray powder diffraction data have been reported previously (*a* = 6.983 Å, *b* = 8.911 Å, *c* = 5.675 Å, space group *Pbnm*; Evans *et al.*, 1964 ▸). Nitro­sonium tetra­fluorido­borate crystallizes in the baryte (BaSO_4_) structure type and is isotypic with ammonium, alkali metal (K, Rb, Cs) (Clark & Lynton, 1969 ▸) and di­oxy­gen(1+) tetra­fluorido­borates (Wilson *et al.*, 1971 ▸). The current unit cell (Table 1 ▸) refined from single-crystal X-ray data at 150 K is in good agreement with the aforementioned previously published values.

**Figure 1:**
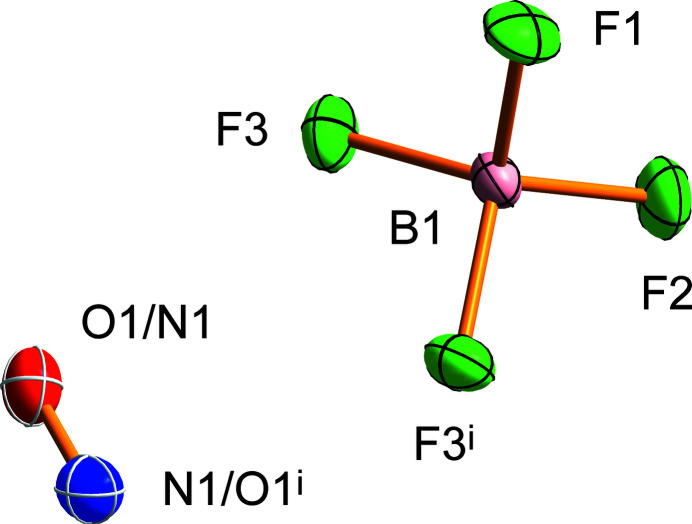
Expanded asymmetric unit of NOBF_4_ with the atom-labelling scheme. Displacement ellipsoids are drawn at the 50% probability level. [Symmetry code: (i) *x*, −*y* + 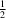 , *z*.]

**Figure 2:**
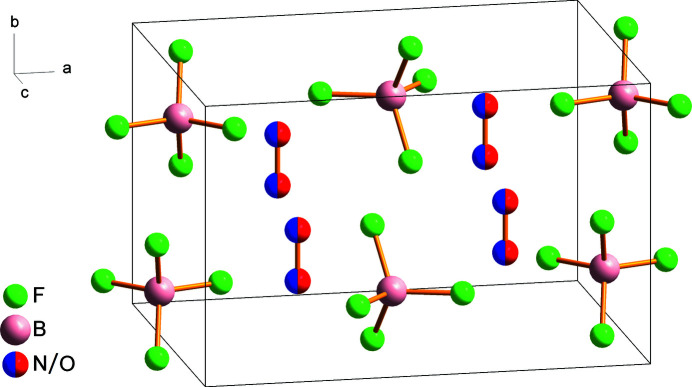
View of the packing in the unit cell of the NOBF_4_ crystal structure.

The asymmetric unit of the NOBF_4_ crystal structure is composed of atoms B1, F1, and F2, which coincide with the crystallographic mirror plane (Wyckoff position 4*c*; site symmetry .*m*.), whereas atoms F3 and disordered N1/O1, are located on general positions (Wyckoff position 8*d*) (Fig. 1 ▸). The BF_4_^−^ anion has a slightly distorted tetra­hedral shape, with F—B—F bond angles ranging from 108.42 (6) to 111.11 (7)° and B—F bond lengths of 1.3863 (10), 1.3872 (10) and 1.4042 (6) Å involving atoms F1, F2, and F3, respectively. Similar values were observed in NO_2_BF_4_ (Krossing *et al.*, 2007 ▸) and other BF_4_^−^ salts (Radan *et al.*, 2011 ▸; Lozinšek *et al.*, 2009 ▸) or complexes (Tavčar & Žemva, 2005 ▸). The NO^+^ cation is disordered across a crystallographic mirror plane, with atoms N1 and O1 sharing the same site. It is noteworthy that the orientational cationic disorder in the salt NOBF_4_ was studied previously by heat capacity measurements from 10 to 304 K (Callanan *et al.*, 1981 ▸). The N—O bond length of 1.0216 (10) Å in NOBF_4_ is similar to the values reported for other nitro­sonium fluoride salts, for instance: 1.052 (6) Å in NOUF_6_ at 100 K (Scheibe *et al.*, 2016 ▸) and 1.012 (6) Å in NOSbF_6_ at 150 K (Mazej & Goreshnik, 2021 ▸), with both salts also exhibiting disordered NO^+^ groups. Each anion is surrounded by seven cations and *vice versa*, with fourteen (N/O)⋯F contacts shorter than 3.0 Å; the shortest contacts [2.6211 (6), 2.6222 (6), and 2.6560 (6) Å] involve atom F3. In the crystal structure, the NO^+^ cations are oriented parallel to the *b* axis (Fig. 2 ▸).

## Synthesis and crystallization

A sample of NOBF_4_ suitable for single-crystal X-ray diffraction was obtained from a commercial source (Alfa Aesar, 98%). Crystals were placed onto a watch glass and covered with a protective layer of perfluoro­deca­lin (ABCR, AB102850, 98%, *cis* and *trans*) inside an argon-filled glovebox (MBraun, H_2_O < 0.5 ppm). A suitable colorless crystal was selected under a polarizing microscope outside the glovebox, mounted on a MiTeGen Dual Thickness MicroLoop with the aid of Baysilone-Paste, and quickly transferred into a cold nitro­gen stream of the X-ray diffractometer.

## Refinement

Crystal data, data collection and structure refinement details are summarized in Table 1 ▸. The coordinates and anisotropic displacement parameters of the disordered atoms O1 and N1 sharing the same site were constrained to be equal (EXYZ, EADP) and their site occupancy factor set to 0.5.

## Supplementary Material

Crystal structure: contains datablock(s) I. DOI: 10.1107/S2414314621012153/wm4156sup1.cif

Structure factors: contains datablock(s) I. DOI: 10.1107/S2414314621012153/wm4156Isup2.hkl

CCDC reference: 2122392

Additional supporting information:  crystallographic information; 3D view; checkCIF report

## Figures and Tables

**Table 1:** Experimental details

Crystal data	
Chemical formula	NO^+^BF_4_^−^
*M*_r_	116.82
Crystal system, space group	Orthorhombic, *P* *n* *m* *a*
Temperature (K)	150
*a*, *b*, *c* (Å)	8.8588 (3), 5.6268 (2), 6.8460 (2)
*V* (Å^3^)	341.25 (2)
*Z*	4
Radiation type	Mo *K*α
μ (mm^−1^)	0.31
Crystal size (mm)	0.24 × 0.19 × 0.12

Data collection	
Diffractometer	New Gemini, Dual, Cu at home/near, Atlas
Absorption correction	Analytical (*CrysAlis PRO*; Rigaku OD, 2021 ▸)
*T*_min_, *T*_max_	0.948, 0.975
No. of measured, independent and observed [*I* > 2σ(*I*)] reflections	16165, 976, 824
*R*_int_	0.054
(sin θ/λ)_max_ (Å^−1^)	0.864

Refinement	
*R*[*F* ^2^ > 2σ(*F* ^2^)], *wR*(*F* ^2^), *S*	0.025, 0.081, 1.10
No. of reflections	976
No. of parameters	37
Δρ_max_, Δρ_min_ (e Å^−3^)	0.22, −0.28

Computer programs: *CrysAlis PRO* (Rigaku OD, 2021 ▸), *SUPERFLIP* (Palatinus & Chapuis, 2007 ▸; Palatinus & van der Lee, 2008 ▸; Palatinus *et al.*, 2012 ▸), *SHELXL* (Sheldrick, 2015 ▸), *OLEX2* (Dolomanov *et al.*, 2009 ▸), *DIAMOND* (Brandenburg, 2005 ▸) and *publCIF* (Westrip, 2010 ▸).

## full crystallographic data

### Crystal data

**Table:**

NO^+^·BF_4_^−^	*D*_x_ = 2.274 Mg m^−^^3^
*M**_r_* = 116.82	Mo *K*α radiation, λ = 0.71073 Å
Orthorhombic, *P**n**m**a*	Cell parameters from 8015 reflections
*a* = 8.8588 (3) Å	θ = 3.8–37.6°
*b* = 5.6268 (2) Å	µ = 0.31 mm^−^^1^
*c* = 6.8460 (2) Å	*T* = 150 K
*V* = 341.25 (2) Å^3^	Irregular, clear colourless
*Z* = 4	0.24 × 0.19 × 0.12 mm
*F*(000) = 224	

### Data collection

**Table:**

New Gemini, Dual, Cu at home/near, Atlas diffractometer	976 independent reflections
Radiation source: fine-focus sealed X-ray tube, Enhance (Mo) X-ray Source	824 reflections with *I* > 2σ(*I*)
Graphite monochromator	*R*_int_ = 0.054
Detector resolution: 10.6426 pixels mm^-1^	θ_max_ = 37.9°, θ_min_ = 3.8°
ω scans	*h* = −15→15
Absorption correction: analytical (CrysAlisPro; Rigaku OD, 2021)	*k* = −9→9
*T*_min_ = 0.948, *T*_max_ = 0.975	*l* = −11→11
16165 measured reflections	

### Refinement

**Table:**

Refinement on *F*^2^	0 restraints
Least-squares matrix: full	Primary atom site location: iterative
*R*[*F*^2^ > 2σ(*F*^2^)] = 0.025	*w* = 1/[σ^2^(*F*_o_^2^) + (0.045*P*)^2^ + 0.0254*P*] where *P* = (*F*_o_^2^ + 2*F*_c_^2^)/3
*wR*(*F*^2^) = 0.081	(Δ/σ)_max_ < 0.001
*S* = 1.10	Δρ_max_ = 0.22 e Å^−^^3^
976 reflections	Δρ_min_ = −0.28 e Å^−^^3^
37 parameters	

### Special details

**Table:**

Geometry. All esds (except the esd in the dihedral angle between two l.s. planes) are estimated using the full covariance matrix. The cell esds are taken into account individually in the estimation of esds in distances, angles and torsion angles; correlations between esds in cell parameters are only used when they are defined by crystal symmetry. An approximate (isotropic) treatment of cell esds is used for estimating esds involving l.s. planes.

### Fractional atomic coordinates and isotropic or equivalent isotropic displacement parameters (Å^2^)

**Table:**

	*x*	*y*	*z*	*U*_iso_*/*U*_eq_	Occ. (<1)
F1	0.34796 (7)	0.250000	0.97160 (7)	0.02402 (13)	
F2	0.59689 (6)	0.250000	0.88293 (9)	0.02508 (13)	
F3	0.42423 (4)	0.45243 (7)	0.70118 (6)	0.02149 (11)	
B1	0.44886 (9)	0.250000	0.81682 (11)	0.01500 (14)	
O1	0.31375 (5)	0.34078 (9)	0.35238 (7)	0.02274 (12)	0.5
N1	0.31375 (5)	0.34078 (9)	0.35238 (7)	0.02274 (12)	0.5

### Atomic displacement parameters (Å^2^)

**Table:**

	*U*^11^	*U*^22^	*U*^33^	*U*^12^	*U*^13^	*U*^23^
F1	0.0301 (3)	0.0280 (3)	0.0140 (2)	0.000	0.00714 (18)	0.000
F2	0.0191 (2)	0.0290 (3)	0.0272 (3)	0.000	−0.01088 (19)	0.000
F3	0.02134 (18)	0.02461 (19)	0.01852 (18)	0.00146 (13)	−0.00060 (11)	0.00719 (12)
B1	0.0152 (3)	0.0187 (3)	0.0111 (3)	0.000	−0.0013 (2)	0.000
O1	0.0181 (2)	0.0266 (2)	0.0235 (2)	0.00024 (16)	−0.00237 (14)	0.00430 (17)
N1	0.0181 (2)	0.0266 (2)	0.0235 (2)	0.00024 (16)	−0.00237 (14)	0.00430 (17)

### Geometric parameters (Å, º)

**Table:**

F1—B1	1.3863 (10)	F3—B1	1.4042 (6)
F2—B1	1.3872 (10)	O1—N1^i^	1.0216 (10)
			
F1—B1—F2	111.11 (7)	F2—B1—F3^i^	109.32 (4)
F1—B1—F3^i^	109.32 (4)	F2—B1—F3	109.32 (4)
F1—B1—F3	109.31 (4)	F3^i^—B1—F3	108.42 (6)

Symmetry code: (i) *x*, −*y*+1/2, *z*.
